# Role of exosomal proteins in cancer diagnosis

**DOI:** 10.1186/s12943-017-0706-8

**Published:** 2017-08-29

**Authors:** Weihua Li, Chuanyun Li, Tong Zhou, Xiuhong Liu, Xiaoni Liu, Xiuhui Li, Dexi Chen

**Affiliations:** 1 YouAn Hospital, Capital Medical University, Beijing, China; 2 Beijing Institute of Hepatology, Beijing, China; 3 Xinjiang Medical University, Wulumuqi, China; 4 NO.8, xitoutiao,Youan men wai, Fengtai District, Beijing, China

**Keywords:** Exosome, Cancer, Diagnose, Biomarker

## Abstract

Exosomes are emerging as a new type of cancer biomarkers. Exosome is a bilayered nano-sized vesicle secreted by various living cells in all body fluids. Based on the expanding albeit incomplete knowledge of their biogenesis, secretion by cells and cancer cell-specific molecular and genetic contents, exosomes are viewed as promising, clinically-relevant surrogates of cancer progression and response to therapy. Preliminary proteomic, genetic and functional profiling of cancer cell-derived or cancer plasma-derived exosomes confirms their unique characteristics. Alterations in protein or nucleic acid profiles of exosomes in plasma correlate with pathological processes of many diseases including cancer. However, previous studies on exosome application in cancer diagnosis and treatment mainly focussed on miRNAs. With the development of rapid large-scale production, purification, extraction and screening of exosomal contents, exosomal protein application can be explored for early stage cancer diagnosis, monitoring and prognosis evaluation. Here, we summarized the recent developments in application of exosomal proteins for cancer diagnosis.

## Background

Cancer is a major public health problem worldwide. Early cancer diagnosis and general awareness of risk factors for cancer have clearly improved survival. However, there is an urgent need to identify more effective and less invasive surrogate markers, which could guide early diagnosis, choice of therapeutic strategies for individual patients, and accurate estimates of prognosis.

Exosomes are produced by a number of cell types. Exosome secretion was described to originate from haematopoietic cells such as reticulocytes [1–3], B lymphocytes and T cells [1, 4–9], platelets [1, 4, 6–10], mast cells [1, 4, 6–9, 11], dendritic cells [1, 4, 6–8] and macrophages [12, 13]. Exosomes are also produced by the cells of non-haematopoietic origin like epithelial cells (intestinal epithelial cells) [1, 6, 14–21], astrocytes [1, 22], neurons [1, 22], melanocytes [12], mesothelioma cells [6], adipocytes [23], fibroblasts [23] and tumour cells [1, 6, 14, 17–21]. Exosomes have also been identified in most bodily fluids including urine and amniotic fluid [24], blood [25], serum [26, 27], saliva [28, 29], ascites [30], breast milk [31], cerebrospinal fluid [32, 33] and nasal secretion [34, 35]. They play an essential role in intercellular communication by carrying their contents. Exosomes reflect the phenotypic state of the parental cell. Intercellular communication mediated by exosomes not only participates in the regulation of normal physiological processes but also in pathological processes of many diseases, including cancer [36–38].

Due to their presence and stability in most body fluids and resemblance of their contents to parental cells, exosomes have great potential as liquid biopsy specimens for various diseases [39, 40]. In particular, cancer-derived exosomes likely serve as biomarkers for early detection of cancer as they carry the cargo reflective of genetic or signaling alterations in cancer cells of origin [41–43]. The objective of this review was to consider the evidence in support of the potential role of cancer-derived exosomes as biomarkers, which in the near future might facilitate monitoring of cancer progression and its outcome.

### Biogenesis and characteristics of exosomes

**Fig. 1:**
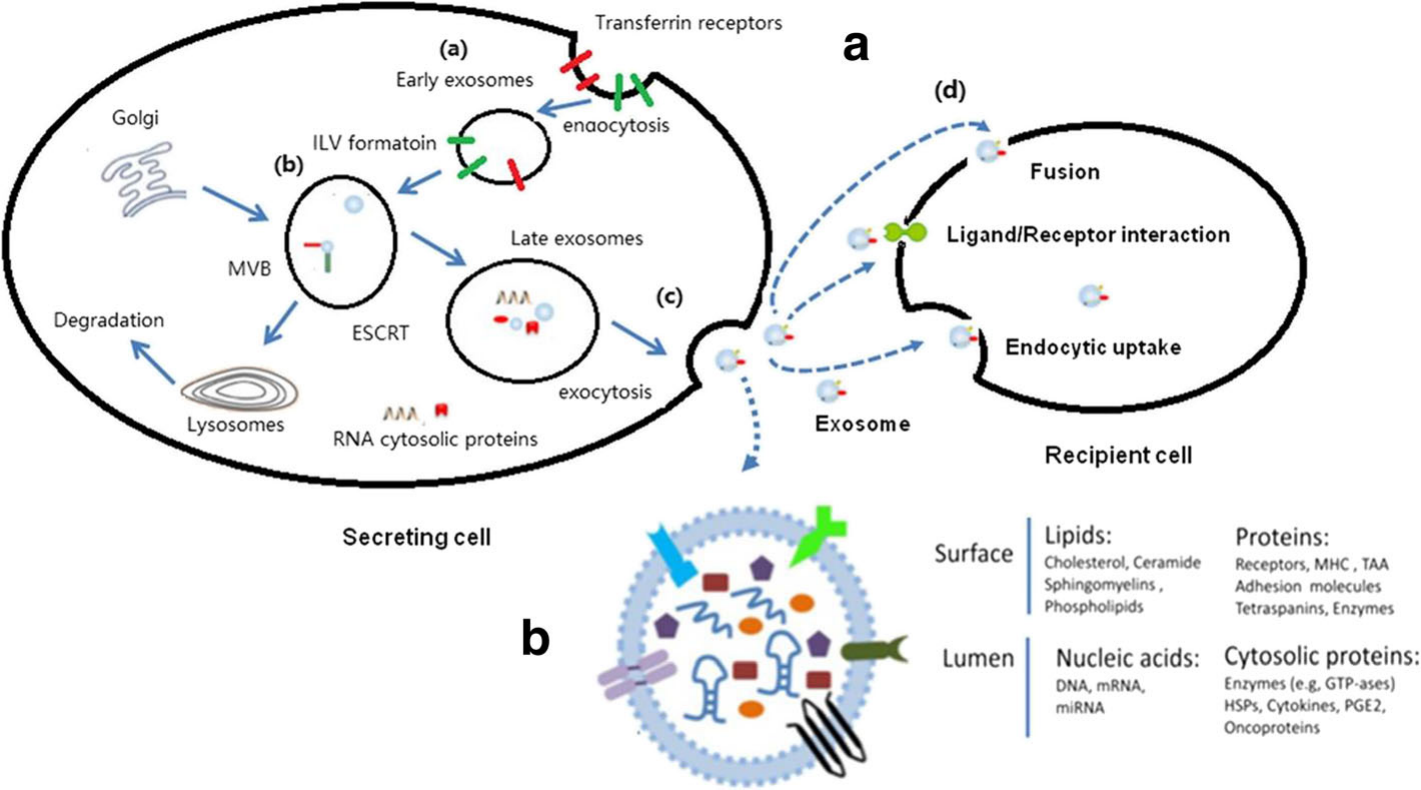
Biogenesis, release, structure, and uptake of exosomes. In **A**, exosomes are repressed by small vesicles of different sizes that are formed as the ILV by budding into early endosomes and multivesicular body and are released by fusion of multivesicular body fuse with lysosomes. (**a**). By endocytosis of membrane segments, the initial endosome arises, containing receptors and transmembrane proteins of the plasma membrane. (**b**). Instead of lysosomal degradation, the matured late endosome transforms by inward budding of tiny vesicles into a multivesicular body. Furthermore, the exosomal cargo as proteins and miRNA, is selectively loaded into the vesicles. (**c**). Exosomes are released into the extracellular space by fusion of the multivesicular body with the plasma membrane. (**d**). Cell-secreted exosomes can travel through biological fluids (e.g. serum, lymph) and be internalized by recipient cells. Exosomes transfer information to the target cells through three main ways: (1) direct fusion with plasma membrane; (2) receptor-ligand interaction; (3) endocytosis by phagocytosis. In **B**, The membrane of the MVBs bulges inward to form exosomes. During this process, proteins (e.g., receptor, cytoplasmic proteins, tetraspanin), nucleic acids (e.g., DNA, mRNA, miRNA), and lipids (e.g., cholesterol, ceramide) are packed into exosomes in a cell type-dependent manner

Exosomes are 40–100 nm extracellular vesicles (EVs), 1.13–1.19 g/mL in density, with a classic “cup” or “dish” morphology [44]. According to the current version of the exosome content database, Exocarta (http://www.exocarta.org), 9769 proteins, 1116 lipids, 3408 mRNAs and 2838 microRNAs have been identified in exosomes of many different cell types and from multiple organism, thus demonstrating their complexity once again [45–47]. The exosomal contents vary between different physiological and pathological conditions and original cell types. Moreover, the composition of exosomes can be distinct from the originated cells due to the selective sorting of the cargo into exosomes. These proteins included constitutive components of exosomes such as tetraspanins (CD9, CD63, CD81, CD82, CD151, Tspan8), [48, 49] cell type-specific molecules such as histocompatibility complex (MHC) class-I and class-II and other molecules such as adhesion molecules, proteases, MHC molecules, HSP60, HSP70 and HSP90, the ESCRT components TSG101 and Alix [44, 50, 51]. In addition to proteins and lipids, exosomes contain large amounts of nucleic acids, such as mRNA, microRNA, circular RNA, long non-coding RNA and DNA, which are protected from degradation due to the double lipid membrane [43, 52–55]. Here, the structure and contents of exosomes is demonstrated in Fig. 1.

Exosome formation is a fine-tuned process which includes four stages: initiation, endocytosis, multivesicular bodies (MVBs) formation, and exosome secretion [44] Multivesicular bodies (MVBs) are endocytic structures formed by the budding of an endosomal membrane into the lumen of the compartment [56]. After vesicular accumulation, the MVBs are either sorted for cargo degradation in the lysosome or released into the extracellular space as exosomes by fusing with the plasma membrane (Fig. 1). The mechanisms underlying the sorting of cargo into the intraluminal vesicles (ILVs) are not yet fully elucidated. Both endosomal sorting complex required for transport (ESCRT)-dependent and independent signals have been suggested to determine the sorting of exosomes [57]. The formation of exosomes has been shown to be controlled by the syndecan heparan sulfate proteoglycans and their cytoplasmic adaptor syntenin [58].

Several mechanisms have been suggested to mediate the uptake of exosomes, including a exosome fusion with the cellular membrane of the recipient cell, leading to the release of the exosomal cargo into the cytoplasm, juxtracrine signaling through receptor-ligand interactions, and endocytosis by phagocytosis (Fig. 1). Although the specific receptors that mediate the uptake of exosomes have not been found, there are several proteins that may act as potential receptors for exosome uptake, such as Tim1/4 for B cells [59] and ICAM-1 for APCs [60].

### Exosome – Functions in cancer

**Fig. 2:**
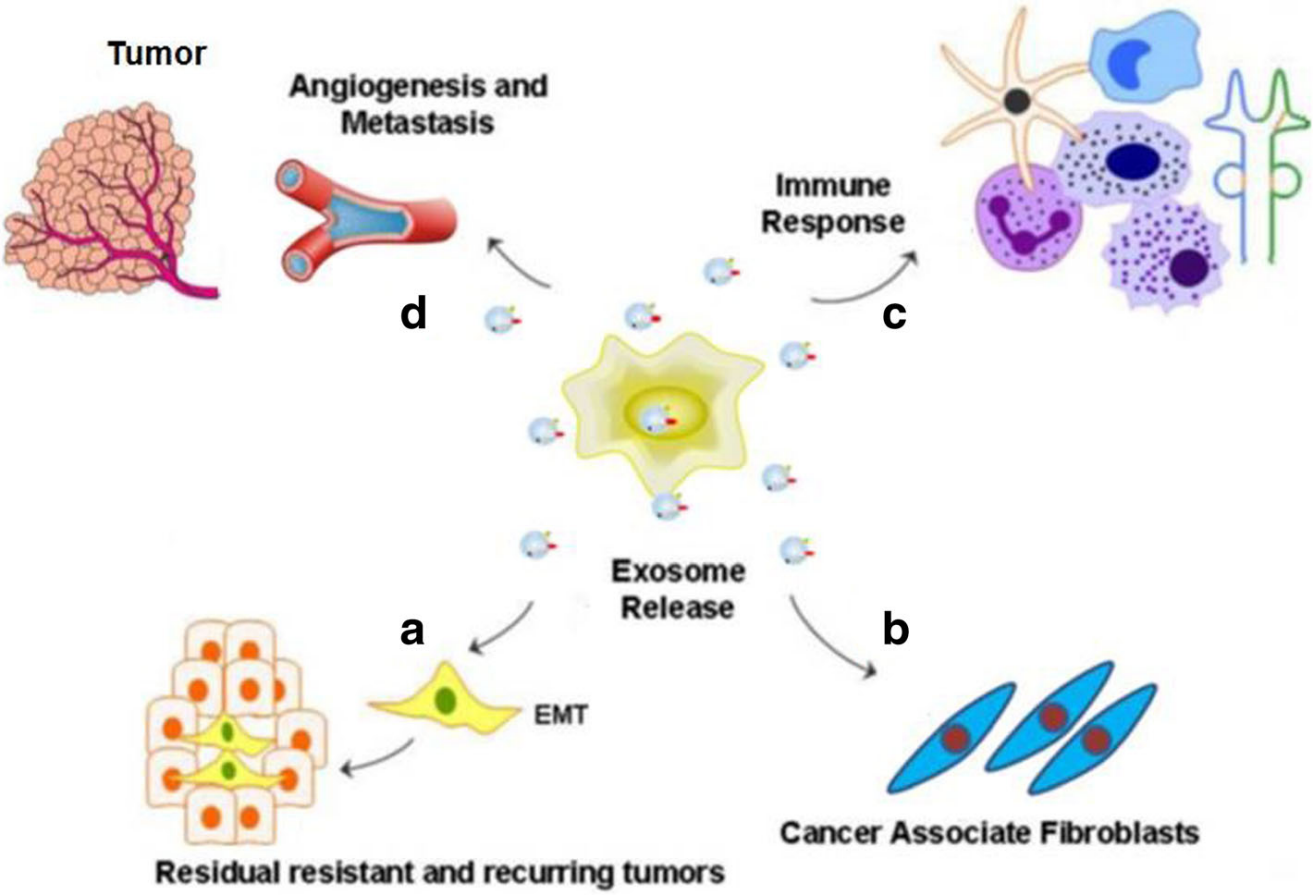
Role of exosomes in sustaining cancer resistance networks. Exosome mediated export of biological material can induce a microenvironment favorable for resistance. Exosome released factor can promote **a** EMT cell morphology, resulting in stemness; **b** promote fibroblast like cell formation that causes desmoplatic reaction (stromal reaction); **c** promote immune escape mechanisms and **d** promote angiogenesis and metastasis. The miRNAs expelled by exosomes can regulate multiple signaling pathways that cumulatively promote resistant phenotype of most tumors

Exosomes play a very important role in cancer progression, metastasis, and therapeutic efficacy. The role of exosomes in cancer development is of particular interest to oncologists because cancer cells secrete at least 10-fold more exosomes than normal cells, Exosomes are involved in initiation, growth, progression and drug-resistance of cancers involving interactions with the microenvironment by transferring oncogenic proteins and nucleic acids [61–64]. First, Exosome promote angiogenesis and metastasis: Exosome uptake induces upregulation of angiogenesis related genes and results in enhanced endothelial cell proliferation, migration, and sprouting [65]. Exosomes derived from hypoxic glioblastoma cells are more potent to induce angiogenesis [66]. Cancer exosomes are responsible for stromal activation [67], induction of the angiogenic switch [68–72], increased vascular permeability [42, 43, 73, 74]. Exosome contribute to metastasis by aiding in the epithelial-tomesenchymal transition and formation of the pre-metastatic niche [75–77]. Second, Exosome contribute to cancer-associated fibroblasts [78–80]. exosomes derived from MDA-MB 231 breast cancer cells and U87 glioblastoma cells were able to induce transformation of recipient fibroblasts, dependent on continuous supply of exosomes [81]. The phenomenon is caused by tissue transglutaminase cross-linked fibronectin (FN) in cancer vesicles, which activates mitogenic signaling. Third, Exosome can lead to immune escape: The generation of an immuno-suppressive environment is an important issue for the pathogenesis of cancers. Exosomes have been shown to be implicated in induction of apoptosis of cytotoxic T-cells, expansion and function of regulatory T-cells (Tregs), induction of M2 polarization of macrophages, inhibition of cytotoxicity of natural killer (NK) cells, inhibition of differentiation of dendritic cells (DC), expansion and activation of myeloid-derived suppressor cells (MDSC) and mobilization of neutrophils [82–85]. Finally, exosomes protect cancer cells from the cytotoxic effects of chemotherapy drugs and transfer chemoresistance properties to nearby cells [86, 87]. Transfer of multi-drug resistant protein Pgp to drug-sensitive cells conferring drug-resistent properties and directing cytotoxic drugs such as cisplatin away from the nucleus have been reported [88, 89] (Fig. 2). Stromal derived exosomes were shown to regulate therapy resistance pathways in breast cancer cells [90]. Expansion of chemotherapy-resistant cancer cells was mediated by activation of the pattern recognition receptor RIG-1, which triggers STAT-1 signaling (antiviral) and induces the interaction of stromal JAG-1 with Notch3 on breast cancer cells [90].

### Potential of exosomal proteins in disease diagnosis and prognosis prediction

Exosome contains abundant proteins. In addition to self-proteins, it also contains proteins derived from cells. Exosomal proteins from cancer cells are becoming new biomarkers for cancer monitoring and efficacy evaluation based on the following characteristics: 1) Cancer-related lipid, protein, RNA and DNA in exosomes can be used to test for cancers [91]. 2) Small volume, strong permeability through body tissue barrier and wide existence in various body fluids make exosomes easily accessible for clinical detection [92].3) Lipid bilayer membrane structure of the exosome protects its contents from degradation by enzymes in blood circulation. Phosphorylation proteins can be separated from exosomal samples frozen for 5 years. 4) The components of blood are complex, and specific proteins secreted by cancer cells are diluted in blood, so a cancer protein will not be easily detected at an early stage or when its amount is lower. In each milliliter of human blood, there are over 10^9^ exosomes. After purification, reasonable numbers of exosomes can be obtained, and cancer-derived exosomes can also be collected. Thus, in vivo detection of exosomes is highly sensitive and conducive to diagnosis of early stage cancer [93]. Based on the above characteristics, detecting exosomal proteins as biomarkers for cancer diagnosis and prognosis evaluation possesses tremendous potential.

### Correlation between exosomal proteins as biomarkers and respiratory disease

#### Lung cancer

**Fig. 3:**
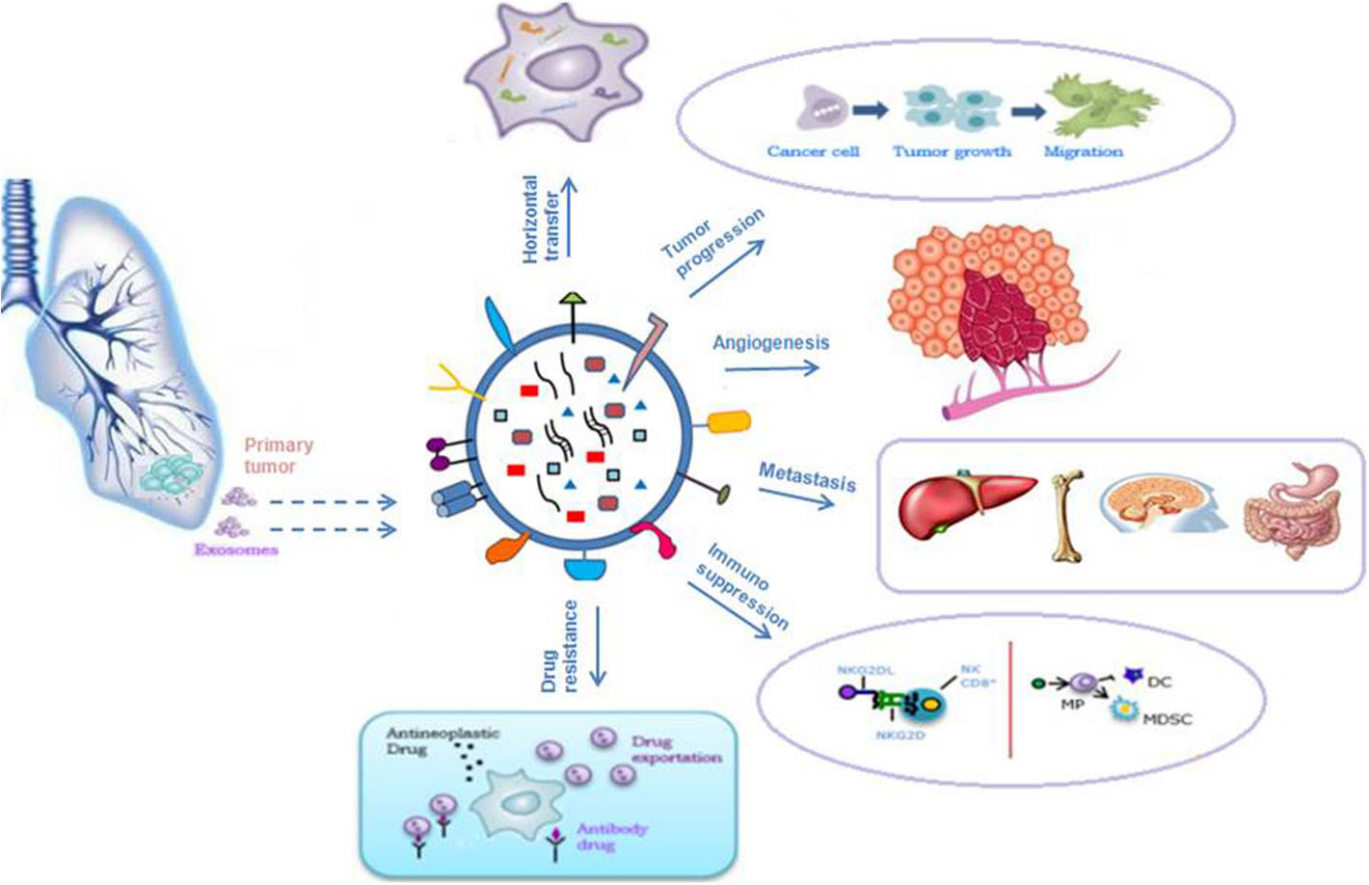
Role of exosomes in NSCLC. Exosomes have a key role in: 1 horizontal transfer of mRNAs and miRNAs from cancer cells to cells of microenvironment; 2 tumor progression, inducing cells motility; 3 angiogenesis; 4: metastatization; 5: immunosuppression; 6: drug resistance

Lung cancer is one of the most common cancers in men and women. An important problem in the diagnosis of lung cancer is the lack of a universal biomarker(s) for early diagnosis. Close to 70% of patients with lung cancer present locally advanced or metastatic disease at the time of diagnosis. Recent studies suggest that exosomes can be potentially useful new tools [94]. Exocrine plays an important role in the occurrence, development and metastasis of lung cancer (Fig. 3). Exosomes secreted by platelets have demonstrated to promote cancer growth and metastasis of lung cancer cells [95]. Grange and colleagues showed that exosomes released from renal cells can promote angiogenesis in lung cancer ascites [96]. That information may be crucial for cancer progression and angiogenesis according to a recent published research showing the involvement of cancer cells-released exosomes in that matter [97] (Fig. 3). Exosomes have been shown to be involved in several cellular functions and intrinsic mechanisms of cancer where they possibly constitute valuable biomarkers [98]. Clark D et al. [99] found that various protein components accumulated in exosomes secreted by lung cancer cells, which could promote the occurrence of lung cancer and facilitate early stage diagnosis. Jakobsen et al. [100] reported that CD317 and epidermal growth factor receptor (EGFR) were highly expressed on exosomal surface, by analyzing the extracellular vesicles secreted by lung cancer cells. These molecules are reliable biomarkers for diagnosing non-small cell lung cancer (NSCLC). Li et al. [101] found that human leucine rich alpha-2-glycoprotein 1 (LRG1) in urinary exosomes was a potential biomarker for diagnosing NSCLC. Proteomic mass spectrometry showed that LRG1 accumulated in urinary exosomes, and was significantly highly expressed in NSCLC patients as compared to healthy individuals. Furthermore, they also reported that LRG1 protein was highly expressed in cancer tissues. Therefore, LRG1 protein in urinary exosomes was derived from cancer tissues. Sandfeld et al. [102] used 49 antibodies to detect exosomal proteins from 431 lung cancer patients and 150 healthy individuals. They found that CD171, CD151 and tetraspanin 8 were significantly highly expressed in patients as compared to healthy individuals. In squamous-cell carcinoma and small-cell lung cancer patients, CD151 is also an independent biomarker. Recently, exosomes in peripheral blood were reported to contain 30 specific biomarkers. Therefore, exosomes and their related components provide a theoretical basis for exploring molecular biomarkers for early stage lung cancer diagnosis.

#### Nasopharyngeal cancer

Keryer et al. [103] first detected latent membrane protein 1 (LMP1) in exosomes from nasopharyngeal cell lines infected with Epstein Barr virus (EBV) in 2006. They found that nasopharyngeal cancer cells could release HLA II and exosomes containing galectin 9 and/or LMP1. LMP1 could inhibit T cell viability. Klibi et al. [104] found exosomes carrying LMP1 in blood and saliva from the nasopharynx of cancer patients. Houali et al. [105] detected LMP1 and BARF1 coded by EBV in serum and saliva from teenagers and adults, and in adult nasopharyngeal cancer patients’ serum and saliva at 62% and 100%, respectively. Animal experiments demonstrated that LMP1 secretion was related to exosomes. Both proteins were good markers for nasopharyngeal cancer diagnosis, especially BARF1, because it covered the entire age range. Their pro-mitotic activities facilitated the occurrence and development of nasopharyngeal cancer. Abundant LMP1 was also detected in exosomes from EBV-infected nasopharyngeal cancer cells [106]. Cancer exosomes could be continuously detected in the plasma of nasopharyngeal cancer patients. Furthermore, the increase in serum exosomal concentration of nasopharyngeal cancer patients was closely related to terminal-stage lymphatic metastasis and poor prognosis [107].

### Correlation between exosomal proteins as biomarkers and digestive system disease

#### Pancreatic cancer

Glypican-1 (GPC1)-positive exosome is a diagnostic index of early stage pancreatic cancer [42]. Circulating exosomes containing GPC1 (GPC1 + Exos) were isolated from blood of 250 pancreatic cancer patients, which helped to distinguish between chronic pancreatitis and pancreatic cancer patients (in early and terminal stages). In animal experiments, GPC1 + Exos in blood were significantly increased, before cancer imaging could be used. Furthermore, GPC1-positive exosomes could also be used as a preoperative and postoperative prognostic index. GPC1 + Exos was a significantly better prognostic marker of pancreatic cancer than CA19–9. Thus, GPC1 + Exos could be used to diagnose pancreatic cancer at early and terminal stages with high accuracy and sensitivity, and as a detection index for therapy. Macrophage migration inhibitory factor (MIF) was found to promote hepatic metastasis of cancers, and could be used as an early stage diagnostic marker for pancreatic cancer hepatic metastasis.

#### Liver cancer and cholangiocarcinoma

A mouse liver damage model was established to analyze urinary exosomal proteomics [93]. Twenty-eight novel exosomes closely related to disease were found, in which CD26, CD81, S1C3A1 and CD10 could be used as markers for hepatic damage. In cholangiocarcinoma model caused by *Opisthorchis viverrini*, 154 proteins were disrupted after cancer onset. To find the specific marker for diagnosing cholangiocarcinoma caused by *Opisthorchis viverrini* in circulating body fluids, exosomes from peripheral circulation of patients were extracted and compared with those from cholangiocarcinoma cell line KKU055. Finally, 27 specific proteins were identified, which provided an experimental basis for cholangiocarcinoma diagnosis.

#### Gastric cancer

**Fig. 4:**
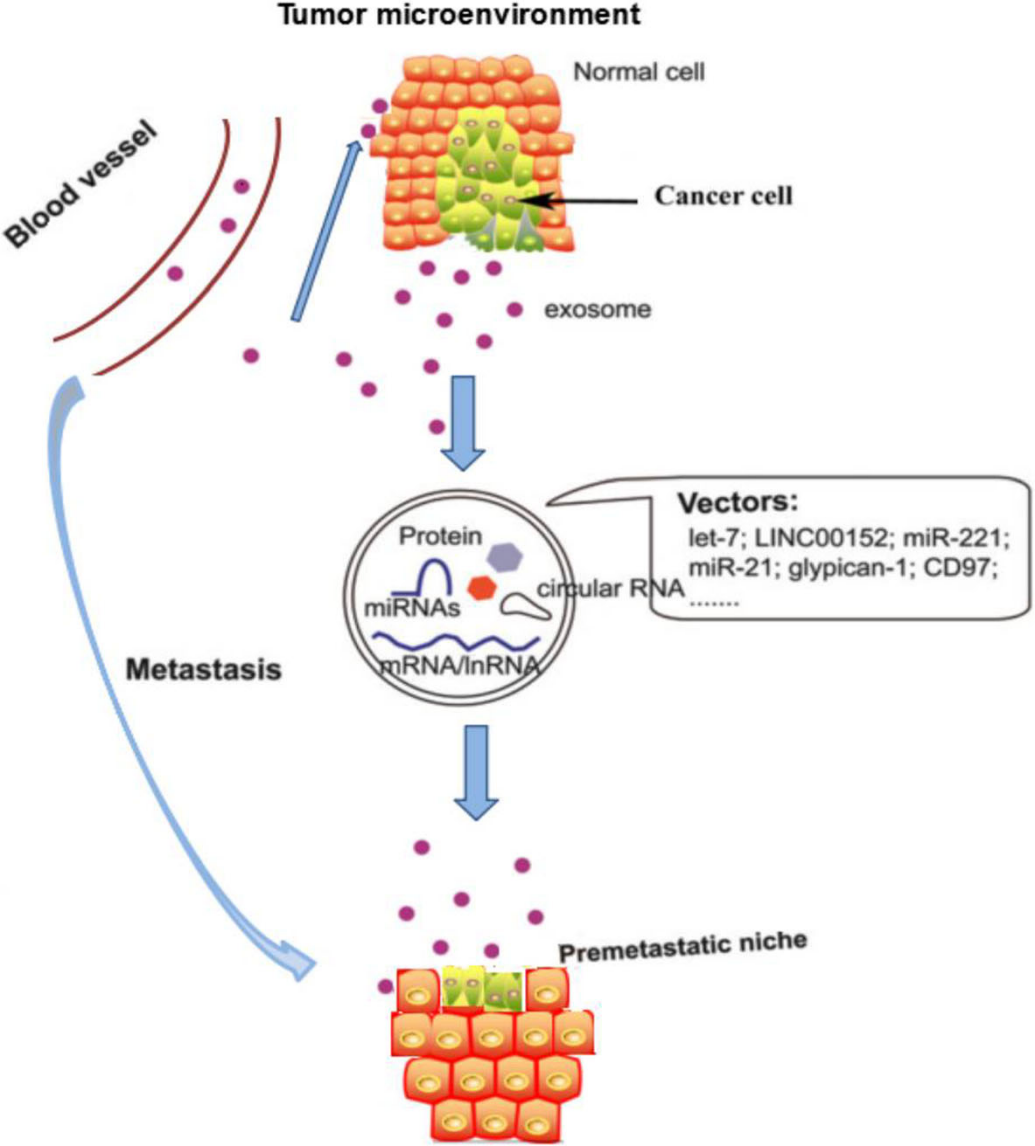
The schematic representation of the role that exosomes play in gastric cancer carcinogenesis and metastasis is summarized in the figure

In 2015, an estimated 24,590 people were diagnosed and 10,720 people eventually died of the disease in the United States [108].^.^As one of the most lethal cancers, gastric cancer (GC) is rampant in many countries around the world. GC is the fourth most common cancer and the second leading cause of cancer death, worldwide [109]. As a carrier, exosomes play an important function in the interaction between cancer cells, the vascular endothelial cells and the macrophages. Exosomes derived from GC cells could also stimulate the activation of the NF-ƙB pathway in macrophages to promote cancer progression [110]. Recent evidence has found that AZ-P7a, a metastatic GC cell line, released let-7 miRNAs via exosomes into the extracellular environment to maintain the oncogenesis [111]. The enrichment of let-7 miRNA family in the exosomes from AZ-P7a cells may reflect metastasis in GC. CD97 promotes GC cell proliferation and invasion in vitro through exosomes-mediated MAPK signaling pathway, and exosomal miRNAs are probably involved in the activation of the CD97-associated pathway [112]. the Cbl family of ubiquitin ligases might be involved in regulation of exosome-induced apoptosis of Jurkat T cells by increasing PI3K proteasome degradation, inactivation of PI3K/ Akt signaling, thus mediating some effects of caspase activation [113]^.^. The role of tetraspanin 8-containing exosomes is associated with cell growth and invasion in GC; tetraspanin 8 is an independent prognostic factor in patients with GC [114]. The schematic representation of the role that exosomes play in GC carcinogenesis and metastasis is summarized in (Fig. 4).

Gu et al. suggested that GC cells triggered the differentiation of human umbilical cordderived mesenchymal stem cells to carcinoma-associated fibroblasts by exosomes-mediated TGF-ß transfer and activation of the TGF-ß/Smad pathway, which may represent a novel mechanism for MSCs-to- CAFs transition in cancer [115]. Furthermore, the Cbl family of ubiquitin ligases might be involved in regulation of exosome-induced apoptosis of Jurkat T cells by increasing PI3K proteasome degradation, inactivation of PI3K/ Akt signaling, thus mediating some effects of caspase activation [116]. Exosomes derived from human mesenchymal stem cells promote GC cell growth and migration via induction of the epithelial-mesenchymal transition and the activation of the Akt pathway [117]. CD97 promotes GC cell proliferation and invasion in vitro through exosomes-mediated MAPK signaling pathway, and exosomal miRNAs are probably involved in the activation of the CD97-associated pathway [118]. The role of tetraspanin 8-containing exosomes is associated with cell growth and invasion in GC; tetraspanin 8 is an independent prognostic factor in patients with GC. Additionally, TEX may play a critical role in the development of peritoneal metastases of GC, which may partially be due to the increased expression of the adhesion molecules fibronectin 1 (FN1) and laminin gamma 1 (LAMC1) in mesothelial cells [39]. The schematic representation of the role that exosomes play in GC carcinogenesis and metastasis is summarized in (Fig. 1).

Baran et al. [119] found that the number of exosomes was significantly higher in gastric cancer patients than in the normal control group. Expressions of human epidermal growth factor receptor (HER-2/neu) and human chemokine receptor-6 (CCR6) were significantly increased on exosomal surface in blood. Cancer markers such as HER-2/neu, melanoma antigen (MAGE) and c-Met as well as extracellular matrix metalloproteinase inducer (EMMPRIN) could be detected in exosomes from normal controls and gastric cancer patients. However, the patients expressed higher levels of these markers, of which MAGE-1 and HER-2/neu mRNA had significantly higher expression in exosomes from gastric cancer patients.

#### Colorectal cancer

Adenomatous polyposis coli (APC) mutation was found in early stage colorectal cancer, invisible chromosome familial adenomatous polyposis and most sporadic colorectal cancers [120], and might lead to its occurrence and development. Comparison between SW480 cells transfecting APC gene plasmid and exosomal proteome secreted by SW480 cells showed that dickkopf-related protein 4 (DKK 4) was highly expressed in exosomes of SW480 cells transfecting APC gene plasmid. In these cells, methylation level of DKK 4 gene promoter was decreased, suggesting that colorectal epithelial cells might up-regulate DKK 4 transcription and expression by down-regulating methylation of DKK 4 gene promoter, and further promote occurrence and development of colorectal cancer by exosomes secreting DKK 4 and inducing APC gene mutation. The comparison of exosomal proteome between KRAS wild type DKS-8 cells of human colon adenocarcinoma cells DLD-1 and K-RAS mutant type DKO-1 cells indicated that DKS-8 secreted by DKO-1 exosomes was not only significantly proliferated but its invasion capacity was also increased. These experiments demonstrated that colorectal cancer exosomes played a critical role in maintaining cancer cell survival, proliferation and invasion of microenvironment. Silva et al. [121] quantitatively detected plasma exosomes from 91 colorectal cancer patients and found that the number of exosomes was significantly higher than in the control group, and also significantly correlated with carcinoembryonic antigen (CEA). Thus, number of plasma exosomes in colorectal cancer patients can be used as a cancer marker that is closely related to disease development and poor prognosis.

### Correlation between exosomal proteins as biomarkers and nervous system disease

#### Parkinson’s disease

Fraser et al. [65] found leucine-rich repeat kinase 2 (LRRK2) as a biomarker in urinary exosomes from Parkinson’s disease patients. They used ratio of ser-1292 LRRK2 to total LRRK2 to predict LRRK2 gene mutation status and Parkinson’s disease risk in carriers of LRRK2 gene mutation, and found that ser-1292 LRRK2 was closely related to Parkinson’s disease. Then they compared ser (P)-1292 LRRK2 levels in urine samples of 79 Parkinson’s disease patients with 79 healthy controls and found that ser (P)-1292 level in urinary exosomes of primary Parkinson’s disease patients was significantly increased as compared to controls, and was closely related to severity of the disease [122].

#### Spongioblastoma

The detection of serum exosomes from 25 spongioblastoma patients indicated the presence of a spongioblastoma-specific epidermal growth factor receptor variant type III (EGFRVII). Thus, detecting exosomes in blood derived from cancers could provide diagnostic information and adjuvant therapy for cancer patients [123]. Furthermore, micro-fluidic chip was used to analyze exosomal protein types in blood circulation of spongioblastoma patients. Exosomes containing EGFR-VII, EGFR, PDPN and IDH1 secreted by spongioblastoma were isolated, which confirmed that detection of circulating exosomes could predict efficacy of clinical drugs and cancer mutations [124].

### Correlation between exosomal proteins as biomarkers and genitourinary system disease

#### Renal cell carcinoma

Raimondo et al. [125] studied exosomes in urine samples from nine patients with renal cell carcinoma and 23 healthy controls, and found that matrix metalloproteinase 9 (MMP9), ceruloplasmin (Cp), podocalyxin (PC), DKK 4, and carbonic anhydrase IX (CAIX) were highly expressed. However, aquaporin-1 (AQP-1), extracellular matrix metalloproteinase inducer (EMMPRIN), neprilysin (CD10), dipeptidase 1 and syntenin-1 were repressed. Application of exosomal proteomics of 10 proteins has potential clinical value for early stage diagnosis of renal cell carcinoma.

#### Bladder cancer

Chen et al. [126] applied isotopic dimethylation labeling coupled with liquid chromatography-tandem mass spectrometry (LC-MS/MS) to identify bladder cancer biomarkers in urinary microparticles isolated from hernia (control) and bladder cancer patients. They identified 2964 proteins based on more than two distinct peptides, of which 2058 had not been previously reported as constituents of human urine exosomes/microparticles. A total of 107 differentially expressed proteins were identified as candidate biomarkers. Differences in the concentrations of 29 proteins (41 signature peptides) were precisely quantified by LC-MRM/MS in 48 urine samples of bladder cancer, hernia, and urinary tract infection/hematuria. Concentrations of 24 proteins changed significantly between bladder cancer and hernia. We quantified cancer-associated calcium-signal transducer 2 (TACSTD2) in raw urine specimens using a commercial ELISA and confirmed its potential value for diagnosis of bladder cancer. Our study revealed a strong association of TACSTD2 with bladder cancer and highlighted the potential of human urinary microparticles in the noninvasive diagnosis of bladder cancer. The expression of CD36 and CD44 in the exosomes was detected by immunoblotting and flow cytometry. The expression of CD36 and CD44 was significantly different between healthy and bladder cancer patients [115].

#### Prostate cancer

Nilsson et al. [127] found that urinary exosomes in patients with prostate cancer expressed β-catenin, prostate cancer gene-3 (PCA-3), transmembrane serine protease 2-ETS transcription factor family member-related gene fusion (TMPRSS2 -ERG) and other prostate cancer-related markers. The expression of prostate specific antigen (PSA) and prostate specific membrane antigen (PSMA) was also detected in urinary exosomes of patients with prostate cancer showing the potential for diagnosis and monitoring of cancer patients.

#### Ovarian cancer

Ovarian cancer is one of the most lethal forms of cancer in women, and until recently, lacked consistent biomarkers useful for its detection. Approximately 70% of cases of ovarian cancer are diagnosed in advanced stage, which has only a 20% survival rate within 5 years of diagnosis. However, if diagnosed at early stage (at Stage I), survival rate is over 90% [67]. Therefore, early diagnosis using exosomal contents may be highly important and may save large number of patients dying from ovarian cancer due to late diagnosis.

Recent discoveries of epithelial cell adhesion molecule (EpCAM) and CD24 in ovarian cancer-derived exosomes have been highly promising alternatives for early detection of ovarian cancer. EpCAM is a glycoprotein that facilitates homo-typical adhesion of cells. It has been found in pseudo-stratified, transitional and simple epithelia on the basolateral surfaces and has been identified as a cargo protein in exosomes.CD24 is recognized as a cancer marker and is associated with poor prognosis of ovarian carcinomas. CD24 can also be found in the cytoplasm inside MVBs and released into the extracellular environment via exosomes, in which case it is correlated with more aggressive forms of ovarian carcinoma, worsening the prognoses and therefore, shortening patients’ survival times [128].In the exosomes isolated from sera of ovarian cancer patients, Li et al. [97] found that epithelial ovarian cancer markers (such as Claudin 4) gradually increased with the development of cancer. Szajnik et al. [129] found that L1CAM, CD24, ADAM10, EMMPRIN, TGFβ1, MAGE3/6 and Claudin-4 in peripheral blood exosomes could facilitate early stage diagnosis of ovarian cancer. Exosomal proteomics research [130, 131] indicated that exosomes in ovarian cancer had abundant cancer occurrence and development-related proteins of the integrin pathway, EGF receptor pathway, Wnt signaling pathway, PI3 kinase pathway, Fgf receptor pathway, Ras, p53 and angiogenic pathway, including phosphorylated and acetylated proteins. These proteins played a very important role in regulating communication between cells and matrix. In depth study of interactions between these molecules and their functions in signal transduction pathways may demonstrate the molecular mechanisms of malignant cancer occurrence and development.

### Correlation between exosomal proteins as biomarkers and other cancers

Exosomal proteins are used as biomarkers in breast cancer cells, melanoma cells and multiple myeloma cells. Melo et al. [132].revealed that miRNA biogenesis in exosomes inhibited the expression of their respective mRNA targets, such as phosphatase and tensin homolog (PTEN) and the transcription factor homeobox D10 (HOXD10), indicating the contribution to breast cancer progression. Persistent increase in CEA and cancer antigen 15–3 levels in exosomes from breast cancer patients is closely related to the number of cancer foci [116]. Comparison of exosomal proteomics between breast cancer cells lines MCF-7 and MDA-MB231 showed that MDA-MB231 exosomes had a high level of matrix metalloproteinase, which was related to its strong metastasis [116]. A study using exosomes in blood to detect occurrence, development and relapse of breast cancer [117] indicated that the number of exosomes increased with cancer invasion ability. Therefore, markers from exosomes may be discovered earlier than biopsy, MRI or breast X ray to detect breast cancer. In serum exosomes from 40 breast cancer patients [118], survivin level was significantly increased, so survivin-2B can be used as a marker for breast cancer diagnosis or prognosis. More specifically, melanomaderived exosomes have been shown to promote metastasis through the preparation of the metastatic niche by the activation of bone marrow-derived progenitor cells [133]. Wolfers et al. [17] found that exosomes secreted by melanoma contained complete cancer antigen, which could activate CD8+ T cells and exhibit anti-cancer activity after being taken up by dendritic cells. Logozzi et al. [134] suggested that CD63 in plasma exosomes could be used as a protein marker for melanoma.

## Prospects

Early stage cancer diagnosis is very important, which can extend lifespan and decrease disease-related death. Exosomes have become a research hotspot as biomarkers for disease diagnosis. On January 21, 2016, the first worldwide cancer diagnostic product based on exosomes was marketed in USA. The liquid biopsy products launched by Exosome Diagnostics marked a new step in exosomal biology [135]. Exosomal membrane and cytomembrane have the same homology. Using a known cancer surface marker antibody fixed on a chip, exosome surface markers in cell and plasma were detected, and about 40% of cancer cell surface markers were found on the exosomal surface [136] suggesting that the detection of exosomal surface markers could partially replace biopsy of cancer cells. As a biomarker, the exosome can provide abundant, stable, sensitive and specific biological information, and is a liquid biopsy specimen with high application value. However, cancer exosomal proteomics research is currently in its nascent stage. Accurate separation, identification and high throughput clinical application of extracellular vesicles are facing huge challenges. Further development of cancer exosomal proteomics as well as improvement of microfluidic techniques for detecting exosomes will certainly improve their usage for cancer diagnosis.

## Conclusions

Exosomes play critical roles in almost all aspects of cancer provide opportunities for the development of exosomes as ideal diagnostic biomarkers and therapeutic targets. They are readily accessible in nearly all body fluids including blood, urine, saliva, and ascites and contain bioactive molecules that reflect the pathological state of the originated cells, thus providing an enriched source of biomarkers. Exosome-shuttled proteins and nucleic acids have been suggested as novel diagnostic and prognostic indicators for a variety of cancers (Table 1).

**Table 1:** Exosomes secreted in different body fluids and their potential cancer markers

Cancer type	Potential Cancer Markers	Source	References
Bladder Cancer	TACSTD2, EDIL-3, Mucin4, EPS8L2, α6-integrin, MUC-1, Basigin	Urine	[126]
Breast cancer	Survivin, Survivin-2B, CEA, Ttumor antigen15-3	Blood	[116, 118]
Colorectal cancer	CEA	Blood	
Lung cancer	EpCAM, EGFR, CEA, LRG-1	Blood	[100, 101, 137]
Melanoma	CD63, Caveolin1, TYRP2, VLA-4, HSP70	Blood	[133, 134, 139]
Nasopharyngeal cancer	LMP1, Galectin-9, BARF-1	Blood, Saliva	[103]
Ovarian cancer	Claudin-4, L1CAM, CD24, ADAM10, EMMPRIN, TGFβ1, MAGE3/6	Blood	[97, 129]
Pancreatic cancer	GPC1, MIF	Blood	[42, 138]
Prostate cancer	Transmembrane, protease, Serine2-ETS, β-catenin, PCA3, PSA, PSMA, ITGA3, ITGB1, survivin, PTEN	Urine	[127, 140]
Renal cell carcinoma	MMP-9, EMMPRIN, Carbonic anhydrase	Urine	[125]
Spongioblastoma	EGFR-VIII, EGFR, PDPN, IDH1	Blood	[123, 124, 139]
Stomach cancer	HER-2/neu, CCR6	Blood	[119]
